# Supplementary material for: Effects of Antiangiogenetic Drugs on Microcirculation and Macrocirculation in Patients with Advanced-Stage Renal Cancer
Source: Cancers (Basel). 2018 Dec 28;11(1):30. doi: 10.3390/cancers11010030 (PMC6357121; doi:10.3390/cancers11010030)
Supplement: Supplementary file 1 [file cancers-11-00030-s001.pdf]

Supplementary Materials: Effects of Antiangiogenetic Drugs on Microcirculation and Macrocirculation in Patients with Advanced-Stage Renal Cancer

Andrea Dalbeni, Chiara Ciccarese, Michele Bevilacqua, Marco Benati, Cristian Caimmi, Luca Cerrito, Federico Famà, Roberto Iacovelli, Anna Mantovani, Francesco Massari Alessandra Meneguzzi, Pietro Minuz, Martina Montagnana, Giovanni Orsolini, Maurizio Rossini, Gianpaolo Tortora, Ombretta Viapiana and Cristiano Fava

Table S1. Main anthropometric/anamnestic characteristics of participants when divided according to the occurrence of a clinically significant increase in BP after TKI initiation.

Parameters		TKI-Sensitive Patients	TKI-Insensitive Patients
Age (years)		65.4 ± 8.7	64.4 ± 9.6
Males (%)		100 (14/14)	66.7 (10/15)
BMI (Kg/m^2)		23.9 ± 3.3	27.3 ± 4.9
Prior Smoke (%)		78.6 (11/14)	53.3 (8/15)
Previous CV disease (%)		42.9 (6/14)	26.4 (4/15)
Antihypertensive drugs (%)	T0	64.3 (9/14)	33.3 (5/15)
	T1	78.6 (11/14)	46.7 (7/15)
	T2	72.7 (8/11)	64.3 (9/15)
	T3	100 (8/8)	76.9 (10/13)

TKI-sensitive, patients with a clinically significant increase in BP (see text); TKI-insensitive, patients without a clinically significant increase in BP (see text); BMI, body mass index; no significant *p*-values emerged comparing every parameter between TKI-sensitive and TKI-insensitive patients, CV, CardioVascular.

Table S2. Comparison of age, BMI, systolic and diastolic BP (in total group and in subgroups defined according to TKI-sensitivity at T1). urinary nitrates. and endothelin-1 between participant taking BP lowering agents and naïve ones at different study. points.

**Parameters**	**Baseline**		**T1**		**T2**		**T3**	
	AHD (14/29)	No AHD (15/29)	AHD (18/29)	No AHD (11/29)	AHD (17/25)	No AHD (8/25)	AHD (18/21)	No AHD (3/21)
Age (years)	66.8 ± 8.6	63.1 ± 9.4	---	---	---	---	---	---
BMI (Kg/m^2)	26.5 ± 4.6	24.8 ± 4.4	---	---	---	---	---	---
SBP (mmHg)	135.7±12.31	137.2 ± 17.4	148.2 ± 17.2	141.1 ± 17.5	137.2 ± 12.1	136.3 ± 10.3	131.9 ± 15.9	138.6 ± 8.1
DBP (mmHg)	83.7±8.9	86.8 ± 11.5	90.0 ± 11.6	84.5 ± 10.2	86.3 ± 8.8	83.8 ± 7.9	80.3 ± 12.0	80.3 ± 13.6
Nitrates/creatinine (μM/μmol of creatinine)	51.2 ± 37.9 §	110.5 ± 87.7 §	91.0 ± 95.1	58.1 ± 42.7	88.5 ± 73.1	66.7 ± 88.8	69.7 ± 52.5	86.8 ± 59.7
ET-1 (ng/mL)	1.68 ± 2.12	1.63 ± 1.88	5.16 ± 1.76	4.73 ± 1.96	---	---	---	---
SBP (mmHg) in TKI-sensitive	132.0 ± 11.1 (9/14) Δ	127.4 ± 16.0 * (5/15)	154.4 ± 11.6 ⁑ (11/18) Δ	153.4 ± 24.2 * (3/11)	133.8 ± 16.6 (8/17)	141.6 ± 7.6 (3/8)	127.3 ± 20.2 (8/18)	--- (0/3)
SBP (mmHg) in TKI-insensitive	142.4 ± 12.7 (5/14)	142.1 ± 16.0 (10/15)	137.0 ± 12.1 ⁑ (7/18)	138.4 ± 16.4 (8/11)	140.2 ± 5.6 (9/17)	133.2 ± 11.2 (5/8)	135.2 ± 12.3 (10/18)	--- (0/3)
DBP (mmHg) in TKI-sensitive	79.6 ± 7.9 ʃ (9/14) Δ	81.8 ± 8.3 (5/15) *	91.7 ± 5.9 (11/18) Δ	95.6 ± 12.1 (3/11) *	83.5 ± 9.8 (8/17)	85.0 ± 8.6 (3/8)	75.4 ± 13.2 (8/18)	--- (0/3)
DBP (mmHg) in TKI-insensitive	91.0 ± 5.5 ʃ (5/14)	89.4 ± 12.3 (10/15)	81.2 ± 14.6 (7/18)	84.0 ± 10.5 (8/11)	88.8 ± 7.4 (9/17)	83.2 ± 8.4 (5/8)	83.7 ± 10.4 (10/18)	--- (0/3)

AHD, antihypertensive drugs assumption, no AHD, not antihypertensive drugs assumption, BMI, body mass index, SBP, systolic blood pressure; DBP, diastolic blood pressure, ET-1, endothelin-1, TKI-sensitive, patients with a clinically significant increase in BP (see text); TKI-insensitive, patients without a clinically significant increase in BP (see text); §, ʃ, ⁑, *: $p < 0.05$; Δ: $p < 0.001$.

Table S3. Comparison of the changes in the main cardiovascular and laboratory parameters in the two subgroups defined according to the occurrence of a clinically significant increase in BP.

**Parameters**	**TKI-Insensitive (14/29)**	**TKI-Insensitive (15/29)**	**TKI-Sensitive (14/29)**	**TKI-Insensitive (15/29)**	**TKI-Sensitive (11/25)**	**TKI-Insensitive (14/25)**
	T1/T0 (n = 29)		T2/T0 (n = 26)		T3/T0 (n = 22)	
ΔSBP (mmHg)	25.3 ± 13.5	−3.8 ± 6.2 §	8.3 ± 17.6	−4.0 ± 14.3	−26.0 ± 61.3	−3.6 ± 19.6
ΔDBP (mmHg)	13.6 ± 7.3	−6.4 ± 8.9 §	5.3 ± 11.4	−3.1 ± 10.1	−19.6 ± 37.8	−5.1 ± 14.7
ΔPlatelets (10^3/μL)	−126.5 ± 153.1	−41.5 ± 73.8 ‡	−130.6 ± 82.9	−11.5 ± 63.6 §	−158.7 ± 218.1	4.8 ± 105.9 ‡
ΔCreatinine (mg/dL)	0.06 ± 0.19	0.07 ± 0.30	0.07 ± 0.23	−0.09 ± 0.24	−0.11 ± 0.25	−0.03 ± 0.22
ΔNitrates/creatinine (μM/μmol of creatinine)	−7.71 ± 37.50	−23.24 ± 88.31	1.80 ± 76.04	−21.67 ± 81.31	−4.00 ± 25.26	−47.61 ± 70.88
ΔBMI (Kg/m^2)	0.45 ± 4.82	−0.72 ± 1.70	−0.21 ± 0.93	−0.62 ± 1.58	−0.35 ± 2.26	−0.35 ± 1.47
ΔCD (10^3/KPa)	2.19 ± 26.79	0.45 ± 9.85	−0.79 ± 11.93	−0.53 ± 10.12	−0.06 ± 11.62	−4.65 ± 6.83

ΔFMD (%)	1.20 ± 3.26	−1.32 ± 10.54	−1.80 ± 5.87	0.06 ± 10.41	−3.82 ± 3.85	−0.72 ± 3.82
ΔcIMT (mm)	−0.07 ± 0.12	−0.03 ± 0.14	−0.09 ± 0.11	0.03 ± 0.14	−0.06 ± 0.07	−0.05 ± 1.13
ΔIndexed LVM (LVM/BSA. g/m^2	12.99 ± 31.93	−4.60 ± 56.56	12.53 ± 28.79	4.52 ± 50.77	3.97 ± 25.82	4.28 ± 38.10
ΔEF (%)	−0.96 ± 9.27	−2.77 ± 8.84	1.16 ± 8.99	−4.23 ± 10.14	−0.45 ± 9.66	−0.17 ± 10.90

TKI-sensitive, patients with a clinically significant increase in BP (see text); TKI-insensitive, patients without a clinically significant increase in BP (see text); SBP, systolic blood pressure; DBP, diastolic blood pressure; CD, carotid distensibility; FMD, flow-mediated dilatation; medium cIMT, carotid intima-media thickness (left and right at average); EF, ejection fraction. *p*: ‡ ≤ 0.05, § ≤ 0.001 (compared to TKI-sensitive patients at the same study points).
